# 8-Hy­droxy-5,7-dimethyl­quinolin-1-ium chloride dihydrate

**DOI:** 10.1107/S1600536812049495

**Published:** 2012-12-08

**Authors:** Kaliyaperumal Thanigaimani, Nuridayanti Che Khalib, Suhana Arshad, Ibrahim Abdul Razak

**Affiliations:** a School of Physics, Universiti Sains Malaysia, 11800 USM, Penang, Malaysia

## Abstract

In the title hydrated salt, C_11_H_12_NO^+^·Cl^−^·2H_2_O, the quinoline ring system is essentially planar, with a maximum deviation of 0.005 (1) Å for all non-H atoms. In the crystal, the three components are linked by O—H⋯O, N—H⋯O, O—H⋯Cl and weak C—H⋯O hydrogen bonds, forming a layer structure parallel to the *ac* plane. The crystal structure is further stabilized by π–π stacking inter­actions, with centroid–centroid distances of 3.5213 (6) and 3.7176 (6) Å.

## Related literature

For background to and the biological activity of quinoline derivatives, see: Balasubramanian & Muthiah (1996*a* ▶,*b* ▶); Morimoto *et al.* (1991 ▶); Markees *et al.* (1970 ▶). For bond-length data, see: Allen *et al.* (1987 ▶). For the stability of the temperature controller used for the data collection, see: Cosier & Glazer (1986 ▶).
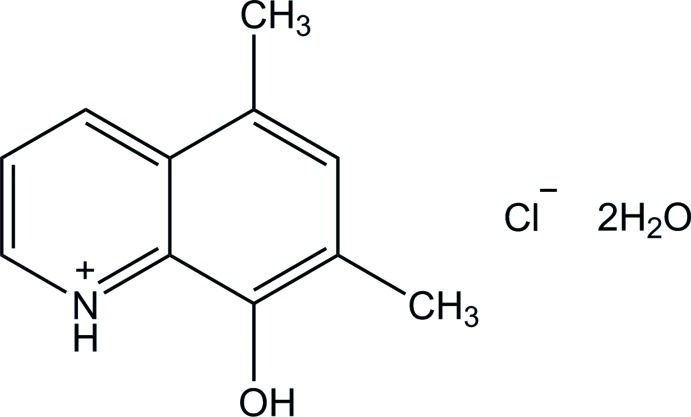


## Experimental

### 

#### Crystal data

C_11_H_12_NO^+^·Cl^−^·2H_2_O
*M* *_r_* = 245.70
Triclinic, 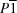

*a* = 6.7990 (5) Å
*b* = 9.2215 (6) Å
*c* = 10.2123 (7) Å
α = 103.820 (1)°
β = 95.629 (1)°
γ = 105.517 (1)°
*V* = 590.04 (7) Å^3^
*Z* = 2
Mo *K*α radiation
μ = 0.32 mm^−1^
*T* = 100 K
0.38 × 0.20 × 0.14 mm

#### Data collection

Bruker APEXII DUO CCD area-detector diffractometer
Absorption correction: multi-scan (*SADABS*; Bruker, 2009 ▶) *T*_min_ = 0.889, *T*_max_ = 0.958
9368 measured reflections
3410 independent reflections
3153 reflections with *I* > 2σ(*I*)
*R*_int_ = 0.018

#### Refinement

*R*[*F* ^2^ > 2σ(*F* ^2^)] = 0.028
*wR*(*F* ^2^) = 0.092
*S* = 1.08
3410 reflections
171 parameters
H atoms treated by a mixture of independent and constrained refinement
Δρ_max_ = 0.43 e Å^−3^
Δρ_min_ = −0.26 e Å^−3^

### 

Data collection: *APEX2* (Bruker, 2009 ▶); cell refinement: *SAINT* (Bruker, 2009 ▶); data reduction: *SAINT*; program(s) used to solve structure: *SHELXTL* (Sheldrick, 2008 ▶); program(s) used to refine structure: *SHELXTL*; molecular graphics: *SHELXTL*; software used to prepare material for publication: *SHELXTL* and *PLATON* (Spek, 2009 ▶).

## Supplementary Material

Crystal structure: contains datablock(s) global, I. DOI: 10.1107/S1600536812049495/is5226sup1.cif

Structure factors: contains datablock(s) I. DOI: 10.1107/S1600536812049495/is5226Isup2.hkl

Additional supplementary materials:  crystallographic information; 3D view; checkCIF report

## Figures and Tables

**Table 1:** Hydrogen-bond geometry (Å, °)

*D*—H⋯*A*	*D*—H	H⋯*A*	*D*⋯*A*	*D*—H⋯*A*
C1—H1*A*⋯O2*W*	0.95	2.55	3.2871 (13)	134
O2*W*—H2*W*2⋯Cl1	0.858 (19)	2.274 (19)	3.1306 (10)	177.4 (18)
O1—H1*O*1⋯O1*W*	0.874 (19)	1.811 (19)	2.6718 (10)	167.9 (18)
N1—H1*N*1⋯O1*W* ^i^	0.826 (18)	1.977 (18)	2.7516 (11)	155.9 (17)
O1*W*—H2*W*1⋯Cl1^i^	0.83 (2)	2.27 (2)	3.0758 (9)	164.0 (18)
O2*W*—H1*W*2⋯Cl1^ii^	0.76 (2)	2.36 (2)	3.1187 (10)	177.7 (18)
O1*W*—H1*W*1⋯O2*W* ^iii^	0.805 (19)	1.86 (2)	2.6690 (11)	177.2 (19)

Symmetry codes: (i) 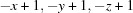; (ii) 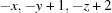; (iii) 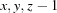.

**Fig. 1.**
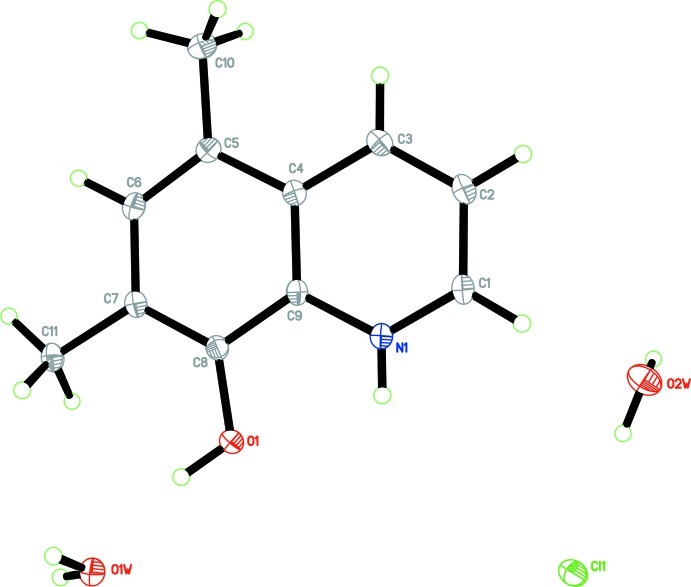
The molecular structure of the title compound with atom labels with 50% probability displacement ellipsoids.

**Fig. 2.**
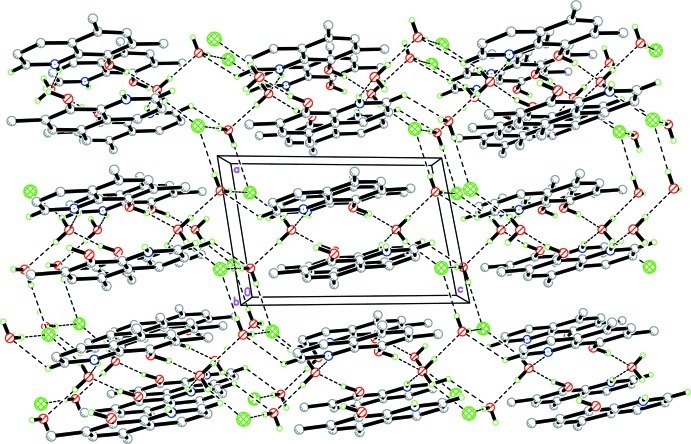
The crystal packing of the title compound. The H atoms not involved in the intermolecular interactions (dashed lines) have been omitted for clarity.

## supplementary crystallographic information

### Comment

Recently, much attention has been devoted to the design and synthesis of supramolecular architectures assembled *via* various weak noncovalent interactions in the crystal structures of oxines (8-hydroxyquinoline), their derivatives and their complexes in a variety of crystalline environments (Balasubramanian & Muthiah, 1996*a*,*b*). Oxine is widely used as analytical reagent. Quinolines and their derivatives are very important compounds because of their wide occurrence in natural products (Morimoto *et al.*, 1991) and biologically active compounds (Markees *et al.*, 1970). In order to study potential hydrogen bonding interactions the crystal structure determination of the title compound (I) was carried out.

The asymmetric unit of the title compound, (I) contains a 8-hydroxy-5,7-dimethylquinolin-1-ium cation, a chloride anion and two water molecules as shown in Fig. 1. One proton is transferred from the hydrochloric acid to the atom N1 of 8-hydroxy-5,7-dimethylquinoline during the crystallization, resulting the formation of salt. The quinoline ring system (N1/C1–C9) is planar with a maximum deviation of 0.005 (1) Å at atom C7. The bond lengths (Allen *et al.*, 1987) and angles are normal.

In the crystal packing (Fig. 2), the ion pairs and water molecules are linked *via* O2W—H2W2···Cl1, O1—H1O1···O1W, N1—H1N1···O1W^i^, O1W—H2W1···Cl1^i^, O2W—H1W2···Cl1^ii^, O1W—H1W1···O2W^iii^ and weak C1—H1A···O2W hydrogen bonds (symmetry codes in Table 1), forming a layer. Furthermore, the crystal structure is stabilized by the following π–π interactions: (*a*) between pyridine (N1/C1–C4/C9, centroid *Cg*1) and benzene (C4–C9, centroid *Cg*2) rings *Cg*1···*Cg*2 (1 - *x*, -*y*, 1 - *z*) 3.5213 (6) Å and (*b*) between benzene rings (C4–C9, centroid *Cg*2) *Cg*2···*Cg*2 (-*x*, -*y*, 1 - *z*) 3.7176 (6) Å.

### Experimental

To a hot methanol solution (20 ml) of 8-hydroxy-5,7-dimethylquinoline (36 mg, Aldrich) was added a few drops of hydrochloric acid. The solution was warmed over a heating magnetic stirrer hotplate for a few minutes. The resulting solution was allowed to cool slowly at room temperature and crystals of the title compound (I) appeared after a few days.

### Refinement

O- and N-bound H atoms were located in a difference Fourier map and were refined freely [O—H = 0.76 (2)–0.873 (19) Å and N—H = 0.828 (19) Å]. The rest of the hydrogen atoms were positioned geometrically (C—H = 0.95–0.98 Å) and were refined using a riding model, with *U*_iso_(H) = 1.2*U*_eq_(C) or 1.5*U*_eq_(methyl C). A rotating-group model was used for the methyl group. Eight outliers were omitted (-3 1 0, 4 -6 2, -4 0 5, -4 -2 3, -4 -3 2, -4 1 5, -4 -3 3 and 3 4 1) in the final refinement.

### Crystal data

**Table:**

C_11_H_12_NO^+^·Cl^−^·2H_2_O	*Z* = 2
*M**_r_* = 245.70	*F*(000) = 260
Triclinic, *P*1	*D*_x_ = 1.383 Mg m^−^^3^
Hall symbol: -P 1	Mo *K*α radiation, λ = 0.71073 Å
*a* = 6.7990 (5) Å	Cell parameters from 6250 reflections
*b* = 9.2215 (6) Å	θ = 2.7–30.1°
*c* = 10.2123 (7) Å	µ = 0.32 mm^−^^1^
α = 103.820 (1)°	*T* = 100 K
β = 95.629 (1)°	Block, yellow
γ = 105.517 (1)°	0.38 × 0.20 × 0.14 mm
*V* = 590.04 (7) Å^3^	

### Data collection

**Table:**

Bruker APEXII DUO CCD area-detector diffractometer	3410 independent reflections
Radiation source: fine-focus sealed tube	3153 reflections with *I* > 2σ(*I*)
Graphite monochromator	*R*_int_ = 0.018
φ and ω scans	θ_max_ = 30.1°, θ_min_ = 2.1°
Absorption correction: multi-scan (*SADABS*; Bruker, 2009)	*h* = −9→9
*T*_min_ = 0.889, *T*_max_ = 0.958	*k* = −12→12
9368 measured reflections	*l* = −14→14

### Refinement

**Table:**

Refinement on *F*^2^	Primary atom site location: structure-invariant direct methods
Least-squares matrix: full	Secondary atom site location: difference Fourier map
*R*[*F*^2^ > 2σ(*F*^2^)] = 0.028	Hydrogen site location: inferred from neighbouring sites
*wR*(*F*^2^) = 0.092	H atoms treated by a mixture of independent and constrained refinement
*S* = 1.08	*w* = 1/[σ^2^(*F*_o_^2^) + (0.058*P*)^2^ + 0.1137*P*] where *P* = (*F*_o_^2^ + 2*F*_c_^2^)/3
3410 reflections	(Δ/σ)_max_ = 0.001
171 parameters	Δρ_max_ = 0.43 e Å^−^^3^
0 restraints	Δρ_min_ = −0.26 e Å^−^^3^

### Special details

**Table:**

Experimental. The crystal was placed in the cold stream of an Oxford Cryosystems Cobra open-flow nitrogen cryostat (Cosier & Glazer, 1986) operating at 100.0 (1) K.
Geometry. All e.s.d.'s (except the e.s.d. in the dihedral angle between two l.s. planes) are estimated using the full covariance matrix. The cell e.s.d.'s are taken into account individually in the estimation of e.s.d.'s in distances, angles and torsion angles; correlations between e.s.d.'s in cell parameters are only used when they are defined by crystal symmetry. An approximate (isotropic) treatment of cell e.s.d.'s is used for estimating e.s.d.'s involving l.s. planes.
Refinement. Refinement of *F*^2^ against ALL reflections. The weighted *R*-factor *wR* and goodness of fit *S* are based on *F*^2^, conventional *R*-factors *R* are based on *F*, with *F* set to zero for negative *F*^2^. The threshold expression of *F*^2^ > σ(*F*^2^) is used only for calculating *R*-factors(gt) *etc*. and is not relevant to the choice of reflections for refinement. *R*-factors based on *F*^2^ are statistically about twice as large as those based on *F*, and *R*- factors based on ALL data will be even larger.

### Fractional atomic coordinates and isotropic or equivalent isotropic displacement parameters (Å^2^)

**Table:**

	*x*	*y*	*z*	*U*_iso_*/*U*_eq_	
Cl1	0.22874 (4)	0.70417 (3)	0.91682 (2)	0.01867 (8)	
O1W	0.49841 (12)	0.45266 (8)	0.25460 (7)	0.01707 (15)	
O1	0.34288 (12)	0.31466 (8)	0.43983 (7)	0.01753 (15)	
O2W	0.22470 (13)	0.42047 (10)	1.03561 (8)	0.02223 (16)	
N1	0.35726 (12)	0.22535 (9)	0.66748 (8)	0.01260 (15)	
C1	0.36928 (14)	0.19382 (11)	0.78767 (9)	0.01493 (17)	
H1A	0.4133	0.2770	0.8699	0.018*	
C2	0.31746 (15)	0.03900 (11)	0.79353 (9)	0.01541 (17)	
H2A	0.3259	0.0159	0.8793	0.018*	
C3	0.25385 (14)	−0.08005 (11)	0.67341 (9)	0.01452 (17)	
H3A	0.2176	−0.1858	0.6769	0.017*	
C4	0.24166 (13)	−0.04734 (10)	0.54510 (9)	0.01229 (16)	
C5	0.17880 (14)	−0.16484 (10)	0.41697 (9)	0.01365 (17)	
C6	0.17410 (14)	−0.11634 (10)	0.29989 (9)	0.01398 (17)	
H6A	0.1328	−0.1942	0.2143	0.017*	
C7	0.22731 (13)	0.04326 (10)	0.29896 (9)	0.01296 (17)	
C8	0.29038 (13)	0.15788 (10)	0.42249 (9)	0.01246 (16)	
C9	0.29632 (13)	0.11195 (10)	0.54492 (9)	0.01168 (16)	
C10	0.11810 (16)	−0.33626 (11)	0.41114 (10)	0.01908 (19)	
H10A	0.0812	−0.3994	0.3153	0.029*	
H10B	−0.0012	−0.3613	0.4573	0.029*	
H10C	0.2348	−0.3595	0.4569	0.029*	
C11	0.21195 (15)	0.08368 (11)	0.16520 (9)	0.01676 (18)	
H11A	0.1307	0.1572	0.1680	0.025*	
H11B	0.1439	−0.0118	0.0908	0.025*	
H11C	0.3513	0.1320	0.1494	0.025*	
H1N1	0.389 (3)	0.318 (2)	0.6664 (18)	0.037 (4)*	
H2W1	0.584 (3)	0.411 (2)	0.2240 (19)	0.040 (5)*	
H2W2	0.228 (3)	0.497 (2)	1.001 (2)	0.050 (5)*	
H1O1	0.385 (3)	0.347 (2)	0.3707 (19)	0.038 (4)*	
H1W2	0.115 (3)	0.390 (2)	1.0495 (18)	0.036 (4)*	
H1W1	0.416 (3)	0.446 (2)	0.1895 (19)	0.038 (4)*	

### Atomic displacement parameters (Å^2^)

**Table:**

	*U*^11^	*U*^22^	*U*^33^	*U*^12^	*U*^13^	*U*^23^
Cl1	0.02078 (13)	0.01844 (12)	0.01979 (13)	0.00704 (9)	0.00619 (9)	0.00844 (9)
O1W	0.0219 (3)	0.0155 (3)	0.0134 (3)	0.0053 (3)	0.0038 (3)	0.0035 (2)
O1	0.0275 (4)	0.0127 (3)	0.0126 (3)	0.0050 (3)	0.0048 (3)	0.0046 (2)
O2W	0.0205 (4)	0.0251 (4)	0.0262 (4)	0.0094 (3)	0.0057 (3)	0.0130 (3)
N1	0.0138 (3)	0.0131 (3)	0.0105 (3)	0.0039 (3)	0.0020 (3)	0.0027 (3)
C1	0.0157 (4)	0.0183 (4)	0.0103 (4)	0.0050 (3)	0.0021 (3)	0.0032 (3)
C2	0.0165 (4)	0.0192 (4)	0.0119 (4)	0.0058 (3)	0.0029 (3)	0.0061 (3)
C3	0.0146 (4)	0.0160 (4)	0.0143 (4)	0.0048 (3)	0.0033 (3)	0.0061 (3)
C4	0.0112 (4)	0.0142 (4)	0.0120 (4)	0.0044 (3)	0.0021 (3)	0.0040 (3)
C5	0.0134 (4)	0.0131 (4)	0.0140 (4)	0.0040 (3)	0.0025 (3)	0.0029 (3)
C6	0.0140 (4)	0.0147 (4)	0.0118 (4)	0.0040 (3)	0.0019 (3)	0.0015 (3)
C7	0.0123 (4)	0.0158 (4)	0.0110 (4)	0.0046 (3)	0.0022 (3)	0.0038 (3)
C8	0.0133 (4)	0.0134 (4)	0.0114 (4)	0.0044 (3)	0.0028 (3)	0.0041 (3)
C9	0.0108 (3)	0.0142 (4)	0.0100 (4)	0.0041 (3)	0.0022 (3)	0.0028 (3)
C10	0.0241 (5)	0.0128 (4)	0.0187 (4)	0.0039 (3)	0.0031 (4)	0.0033 (3)
C11	0.0208 (4)	0.0188 (4)	0.0100 (4)	0.0052 (3)	0.0015 (3)	0.0040 (3)

### Geometric parameters (Å, º)

**Table:**

O1W—H2W1	0.828 (19)	C4—C9	1.4160 (12)
O1W—H1W1	0.806 (19)	C4—C5	1.4262 (12)
O1—C8	1.3558 (11)	C5—C6	1.3735 (12)
O1—H1O1	0.873 (19)	C5—C10	1.5079 (13)
O2W—H2W2	0.86 (2)	C6—C7	1.4211 (12)
O2W—H1W2	0.76 (2)	C6—H6A	0.9500
N1—C1	1.3271 (11)	C7—C8	1.3809 (12)
N1—C9	1.3686 (11)	C7—C11	1.5006 (12)
N1—H1N1	0.828 (19)	C8—C9	1.4129 (12)
C1—C2	1.3935 (13)	C10—H10A	0.9800
C1—H1A	0.9500	C10—H10B	0.9800
C2—C3	1.3768 (13)	C10—H10C	0.9800
C2—H2A	0.9500	C11—H11A	0.9800
C3—C4	1.4127 (12)	C11—H11B	0.9800
C3—H3A	0.9500	C11—H11C	0.9800
			
H2W1—O1W—H1W1	106.5 (17)	C7—C6—H6A	118.0
C8—O1—H1O1	116.0 (11)	C8—C7—C6	118.69 (8)
H2W2—O2W—H1W2	107.8 (19)	C8—C7—C11	121.56 (8)
C1—N1—C9	123.25 (8)	C6—C7—C11	119.75 (8)
C1—N1—H1N1	118.5 (12)	O1—C8—C7	126.20 (8)
C9—N1—H1N1	118.2 (12)	O1—C8—C9	115.02 (8)
N1—C1—C2	120.13 (8)	C7—C8—C9	118.75 (8)
N1—C1—H1A	119.9	N1—C9—C8	118.85 (8)
C2—C1—H1A	119.9	N1—C9—C4	118.90 (8)
C3—C2—C1	119.17 (8)	C8—C9—C4	122.25 (8)
C3—C2—H2A	120.4	C5—C10—H10A	109.5
C1—C2—H2A	120.4	C5—C10—H10B	109.5
C2—C3—C4	121.00 (8)	H10A—C10—H10B	109.5
C2—C3—H3A	119.5	C5—C10—H10C	109.5
C4—C3—H3A	119.5	H10A—C10—H10C	109.5
C3—C4—C9	117.54 (8)	H10B—C10—H10C	109.5
C3—C4—C5	123.88 (8)	C7—C11—H11A	109.5
C9—C4—C5	118.58 (8)	C7—C11—H11B	109.5
C6—C5—C4	117.73 (8)	H11A—C11—H11B	109.5
C6—C5—C10	121.45 (8)	C7—C11—H11C	109.5
C4—C5—C10	120.82 (8)	H11A—C11—H11C	109.5
C5—C6—C7	123.99 (8)	H11B—C11—H11C	109.5
C5—C6—H6A	118.0		
			
C9—N1—C1—C2	−0.37 (14)	C11—C7—C8—O1	0.57 (14)
N1—C1—C2—C3	−0.04 (14)	C6—C7—C8—C9	−1.01 (13)
C1—C2—C3—C4	0.35 (14)	C11—C7—C8—C9	178.35 (8)
C2—C3—C4—C9	−0.27 (13)	C1—N1—C9—C8	−179.47 (8)
C2—C3—C4—C5	179.45 (8)	C1—N1—C9—C4	0.44 (13)
C3—C4—C5—C6	−179.96 (8)	O1—C8—C9—N1	−1.49 (12)
C9—C4—C5—C6	−0.25 (12)	C7—C8—C9—N1	−179.51 (8)
C3—C4—C5—C10	0.53 (13)	O1—C8—C9—C4	178.60 (8)
C9—C4—C5—C10	−179.75 (8)	C7—C8—C9—C4	0.59 (13)
C4—C5—C6—C7	−0.21 (13)	C3—C4—C9—N1	−0.12 (12)
C10—C5—C6—C7	179.30 (8)	C5—C4—C9—N1	−179.85 (8)
C5—C6—C7—C8	0.86 (14)	C3—C4—C9—C8	179.79 (8)
C5—C6—C7—C11	−178.51 (9)	C5—C4—C9—C8	0.06 (13)
C6—C7—C8—O1	−178.78 (8)		

### Hydrogen-bond geometry (Å, º)

**Table:**

*D*—H···*A*	*D*—H	H···*A*	*D*···*A*	*D*—H···*A*
C1—H1*A*···O2*W*	0.95	2.55	3.2871 (13)	134
O2*W*—H2*W*2···Cl1	0.858 (19)	2.274 (19)	3.1306 (10)	177.4 (18)
O1—H1*O*1···O1*W*	0.874 (19)	1.811 (19)	2.6718 (10)	167.9 (18)
N1—H1*N*1···O1*W*^i^	0.826 (18)	1.977 (18)	2.7516 (11)	155.9 (17)
O1*W*—H2*W*1···Cl1^i^	0.83 (2)	2.27 (2)	3.0758 (9)	164.0 (18)
O2*W*—H1*W*2···Cl1^ii^	0.76 (2)	2.36 (2)	3.1187 (10)	177.7 (18)
O1*W*—H1*W*1···O2*W*^iii^	0.805 (19)	1.86 (2)	2.6690 (11)	177.2 (19)

Symmetry codes: (i) −*x*+1, −*y*+1, −*z*+1; (ii) −*x*, −*y*+1, −*z*+2; (iii) *x*, *y*, *z*−1.
